# Correction to: Intraclonal Complexity in Chronic Lymphocytic Leukemia: Fractions Enriched in Recently Born/Divided and Older/Quiescent Cells

**DOI:** 10.1186/s10020-022-00461-0

**Published:** 2022-03-15

**Authors:** Carlo Calissano, Rajendra N. Damle, Sonia Marsilio, Xiao-Jie Yan, Sophia Yancopoulos, Gregory Hayes, Claire Emson, Elizabeth J. Murphy, Marc K. Hellerstein, Cristina Sison, Matthew S. Kaufman, Jonathan E. Kolitz, Steven L. Allen, Kanti R. Rai, Ivana Ivanovic, Igor M. Dozmorov, Sergio Roa, Matthew D. Scharff, Wentian Li, Nicholas Chiorazzi

**Affiliations:** 1 The Feinstein Institute for Medical Research, North Shore–LIJ Health System, Manhasset, NY USA; 2 Department of Medicine, North Shore University Hospital, North Shore–LIJ Health System, Manhasset, NY USA; 3 KineMed, Inc., Emeryville, CA USA; 4 Department of Medicine, University of California, San Francisco, CA USA; 5 Biostatistics Unit, The Feinstein Institute for Medical Research, North Shore–LIJ Health System, Manhasset, NY USA; 6 Department of Medicine, Long Island Jewish Medical Center, North Shore–LIJ Health System, New Hyde Park, NY USA; 7 Oklahoma Medical Research Foundation, Oklahoma City, OK USA; 8 Department of Cell Biology, Albert Einstein College of Medicine, Bronx, NY USA; 9 Department of Molecular Medicine, Hofstra North Shore–LIJ School of Medicine, Hempstead, NewYork USA; 10 Hofstra North Shore–LIJ School of Medicine, Hempstead, NY USA

## Correction to: Molecular Medicine (2011) 17:1374–1382 https://doi.org/10.2119/molmed.2011.00360

Following publication of the original article (Calissano et al. 2011), we have been informed that the legend of Fig. 3 is incorrect.

The corrected text for the Fig. 3 legend is:

Fig. 3 Extended immunophenotype of proliferative and resting CLL fractions. Surface markers were analyzed by flow cytometry in CXCR4^dim^CD5^bright^ (black) and CXCR4^bright^CD5^dim^ (white) fractions. All surface molecules depicted, but CCR7 and CTLA4, were greater in CXCR4^dim^CD5^bright^ fractions, in terms of percent positive cells (**A**) or mean fluorescence intensity (**B**) or of both. CCR7 and CTLA4 were higher in CXCR4^bright^CD5^dim^ fraction. * P < 0.01 and § P < 0.05 (Paired Student t test)
